# The Influence of New Ventures' Work Environment and Innovation Behaviors on Corporate Performance

**DOI:** 10.3389/fpsyg.2022.829037

**Published:** 2022-05-03

**Authors:** Yonghui Xiang, Weiwei Wang

**Affiliations:** ^1^School of Economics and Management, Zhejiang University of Science and Technology, Hangzhou, China; ^2^College of Media and International Culture, Zhejiang University, Hanghzou, China

**Keywords:** new ventures, working environment, innovation behavior, performance, personality psychology

## Abstract

The purpose of the study is to improve the business performance of new ventures. The influence of working environment and innovation behavior on business performance based on personality psychology is studied. First, the relevant theories of new ventures are introduced, and then the structural equation model is displayed. Second, the conceptual model is constructed in response to the two influencing factors of new ventures. Finally, the influencing factors of new venture performance are extracted according to the conceptual model, and a questionnaire is designed. Statistical Product and Service Solutions software is used to analyze the questionnaire data. The results show that the proportion of new enterprise developers is from 8 to 16%, and the number of employees is <150 people. The establishment time of the surveyed enterprises is from 3 to 7 years, and the proportion of the enterprises whose establishment time is <3 years is 30.79%. Management means policies, exploratory innovation, and applicability innovation have different effects on the business performance of new ventures. Among them, the management means of managers have the greatest impact on business performance, accounting for 32.57%, followed by the applicability innovation behavior of employees, accounting for 29.47%; the exploratory innovation behavior of employees takes up 26.47%. The policy environment in the industry where the enterprise is located has the smallest impact on business performance. The results of the hypotheses show that exploratory innovation and applicability innovation do not influence each other; working environment and innovation behavior have a great influence on the performance of enterprises; the most influential factor is the management means of managers. This study provides a reference for new ventures to improve their performance based on the working environment and innovation behavior.

## Introduction

Personality psychology studies a person's unique behavior patterns. Personality refers to the individual in the behavior of the internal tendency, which is manifested in the ability, emotion, need, attitude, motivation, interest, values, and temperament in the process of adapting to the environment. Personality psychology plays a huge role in personality research and is currently applied in many fields. Especially in the field of enterprise management, Hu et al. (2019) put forward the view that psychological knowledge can help managers cultivate talents, indicating that it is conducive to enterprise development.

Wu and Wu (2017) pointed out that the working environment had a certain impact on enterprise performance by constructing the relationship model between the working environment of enterprise employees and enterprise performance, and the influence of managers on employees belongs to the scope of the working environment. Work environment refers to the sum of all potential external factors that affect employees' work performance. In addition to the influence of managers, Miao and Cao (2019) found that there are other factors affecting enterprise performance, including national policies and industry status. Rogoza et al. (2018) proposed that in enterprise production, employee emotions often have a huge impact on enterprises. Wu and Wu (2019) found that employees' competitive behavior may improve enterprise performance, which is related to employees' narcissism. Employee's emotions are directly affected by managers. Wu and Song (2019) found the influence of positive emotions on enterprise performance in the research on the influencing factors of enterprise performance. Qian et al. (2018) also proposed that managers' positive emotions indirectly affect enterprise performance.

With the acceleration of world economic globalization, more and more start-ups begin to emerge. Innovation is the core competitiveness of start-ups. Wu W. et al. (2020) proposed that innovation plays a vital role in the development of start-up companies. Chen et al. (2019) found that employees' innovative behavior is the key to improving enterprise performance. The four conditions of trust, profit, learning, and social interaction of entrepreneurial groups. Wu et al. (2019) proposed that employees' learning behavior has a positive impact on enterprise performance. Yuan and Wu (2020) found that trust has an impact on enterprise performance, Wu Y. J. et al. (2020) proposed the importance of learning behavior by influencing the factor model. Zheng et al. (2018) found that employee learning behavior has a positive effect on enterprise performance. Employees' innovative behaviors have a positive effect on enterprises to maintain benign competition. Employees' innovative behaviors are affected by their characters and they show different enthusiasm for innovative behavior. For example, Chen (2019) proposed that a proactive personality has a positive impact on innovative behavior.

How to improve the corporate performance of new ventures is a common problem for Chinese enterprises. Therefore, the impact of the working environment of new ventures on corporate performance is analyzed based on the theories of personality psychology, providing a feasible path for the improvement of the corporate performance of new ventures. Based on the knowledge of relevant theoretical knowledge and theoretical model of new ventures, assumptions and model construction of business performance under the influence of working environment and innovation behavior are made, and the questionnaire is designed. And the structural equation model is introduced to analyze the relationship between the impact indicators. On this basis, the questionnaire design is carried out, and the influence of specific impact indicators is mainly studied. Then, the questionnaire is recovered and the data are analyzed. The model is optimized and revised, and the influence of new ventures on the working environment and innovation behavior is analyzed. The innovation of this study is to use the structural equation model to study the influencing factors of new ventures. The research content has certain reference significance for the improvement of the business performance of new ventures. The conclusions of the study provide certain development directions for new ventures in the construction of working environments and employee innovation behavior.

## Theories and Research Methods of New Ventures

### New Ventures and Enterprise Performance

New venture generally refers to the start-up time of the enterprise is about 7 years. The start-up time of different enterprises is different, and it is usually between 4 and 11 years. Another view is that new ventures refer to enterprises that are still in the development stage in the whole life cycle of enterprises. Enterprises will be influenced by many factors in the process of entering mature enterprises, such as the development status of the industry in which the enterprise is located, the policies of the industry in which the enterprise belongs, and the imperfect internal organizational system of the enterprise (Gao et al., 2020). Compared with mature enterprises, new ventures often show obvious vulnerability and are prone to financial difficulties in resisting risks (Zhang et al., 2021). However, new ventures still have some advantages. For example, due to the low cost of trial and error, new ventures pay more attention to enterprise innovation. At the same time, the simple organizational system makes them more flexible (Feng and Chen, 2020).

Business performance of enterprises refers to the enterprise operating efficiency within a certain operating time. The measurement indicators of business performance include corporate solvency, corporate profitability, and corporate follow-up development capability (Ren et al., 2020). Business performance under the influence of innovation can be divided into process performance and output performance according to the length of the process of innovation participation. Process performance refers to the innovation behavior involved in the whole process of enterprise production performance, and output performance refers to the innovation behavior which influences the performance of product input in the market. Many factors are affecting the performance of start-ups, such as innovative talents, industry market status, corporate profits (Hadi and Santoso, 2020).

Employee working environment refers to the sum of all factors that affect an employee's work efficiency. An employee's working environment consists of various aspects, such as policies, living environments, development prospects, and management means. Shen et al. (2019) found that excessive supervision by managers can cause great pressure on employees, which harms business performance (Shen et al., 2019). Employees' innovative behavior refers to the behavior that employees produce innovative ideas in the process of work, and apply this idea to enterprise production, and finally make achievements. Employee's innovation behavior can be divided into exploratory innovation and applicability innovation according to the purpose of innovation. The influencing factors of employee innovation behavior include personal personality characteristics, personal-psychological state, personal work characteristics, working environment, and managerial behavior (Wang et al., 2019). Employees' working environment and innovation behavior affect employees' work enthusiasm and business performance.

The measurement indicators of the impact of the working environment and innovation behavior of new ventures on business performance are divided into financial indicators and non-financial indicators.

Financial indexes include corporate solvency, profitability, corporate profits, and so on. Non-financial indexes include innovative talents, market conditions, and policy impact.

### Theory of Structural Equation Model

In this study, some indicators are abstract, and they often need to be transformed with multiple observable indicators. The structural equation model can meet the requirement. The structural equation model is a unique indicator analysis model constructed by proposing hypotheses. The characteristic of the structural equation model is that the establishment of the model is always supported by theories, and the number of samples selected by the model is positively correlated with the feasibility of the results (Shi et al., 2020). The structural equation model originated in the 1970s, is a model combined with path analysis and latent variable research. The basic idea of the structural equation model is that the theoretical hypothesis is put forward based on the research question, and then the theoretical model is constructed according to the theoretical hypothesis. The structural equation model is often used in the research of social science problems, and abstract indexes are often used in the research of these problems. It is difficult to observe these indexes, so the general practice is to set one or more observable indexes for abstract indexes, and then study the problem. The structural equation model is a model to establish the relationship between abstract variables and observable variables.

The basic characteristics of the structural equation model include theoretical apriority and applicability to large sample analysis. Theoretical apriority means that the construction of the structural equation model is based on the existing theory. In the operation of the model, model construction, simulation fitting, model correction, and data results need theoretical support. For large sample analysis, the complexity and the variable number of the structural equation model are more than other models, and the construction of the structural equation model also needs large sample data as support. It can be seen from the characteristics of the structural equation model that the model is suitable for the research. In this study, the evaluation indicators of the impact of the working environment and innovation behavior on business performance include the enterprise survival environment, the policy environment of enterprises, the influence of managers, exploratory innovation, and applicability innovation. The correlation of these indicators needs to be analyzed, and the structural equation model is selected to observe the abstract indicators. And on this basis, the questionnaire is designed. The relationship between the indicators is processed by Analysis of Moment Structure software.

### Hypotheses and Modeling

The development of new enterprises is closely related to the policy of the industry. The policies formulated by the government have certain support for new enterprises. In this case, the enthusiasm of enterprise employees will be improved, and then affect enterprise performance. Good management practices, such as the humility of managers mentioned earlier, can promote employee motivation (Nagle, 2020). Employees' innovative behavior improves business performance. Based on the above content, it can be found that corporate performance is related to industrial policies, employees' attitudes, management tools of managers, and employees' innovation behaviors. Because of the influence of employees' working environment and innovation behavior, the hypotheses of this study are given as follows.

H1: the industry policy has a positive effect on business performance.
H2: Good management means play a positive role in business performance.
H3: Employee exploratory innovation has a positive effect on business performance.
H4: Employee's applicability innovation has a positive effect on business performance.
H5: There is a mutual influence among new venture performance, employee applicability innovation, and employee exploratory innovation.

**Figure 1:**
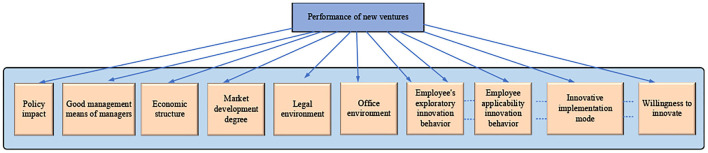
The conceptual model of influencing factors.

According to the hypotheses, the conceptual model of the two influencing factors of new ventures is constructed based on the characteristics of the structural equation model. The conceptual model diagram is shown in Figure 1.

**Figure 2:**
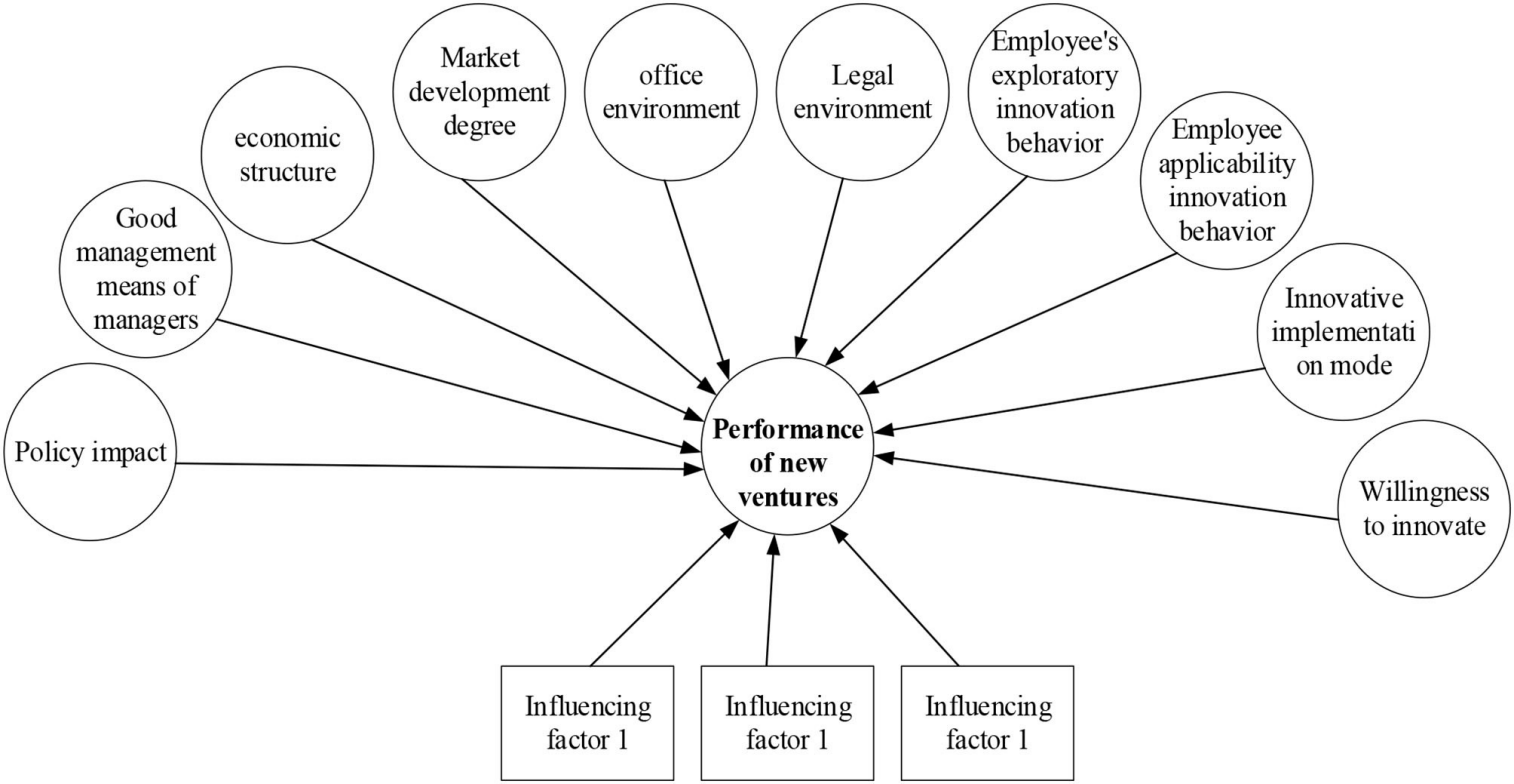
Model of the impact of working environment and innovation behavior on business performance.

Figure 1 shows that the influencing factors of enterprise performance in the model based on the enterprise working environment and innovation behavior are 10, which are the good management mode, policy impact, the economic system, market development degree, legal environment, the office environment, employee exploratory innovation behavior, employee applicability innovation behavior, innovation realization mode, and innovation willingness. Based on the relevant research, if the path coefficient in this model is 1 (Meagher, 2020), the model diagram of the impact of specific working environment and innovation behavior on business performance is given combined with the characteristics of the structural model, as shown in Figure 2.

From the above information, combined with the relevant characteristics of the structural equation model, it is found that among the 10 abstract variables, exploratory innovation and applicability innovation of employees affect each other. And 10 abstract variables directly affect the 11th abstract variable, namely, the performance of new ventures. Each abstract variable has 2–3 specific influencing factors, and the specific index influencing factors will be given later.

The following research involves the optimization of the structural equation model, and the process of model optimization is given here. First, the optimization of the model is carried out by AMOS software. Whether the significance probability of the innovation model is <0.05 is used to judge whether the significance of the model is good or not, and the null hypothesis is rejected. In the study, when the sample size is >150, the significant index of the structural equation model can not be used as the standard of model performance evaluation. The chi-square-freedom ratio is introduced as the measurement index, and the fitting is carried out by AMOS software. Then the model is simplified and corrected by software.

### Questionnaire Design

Based on the relevant literature, the number and feasibility of various indicators are studied by referring to experts' opinions (Pérez-Fuentes et al., 2019; Pinheiro, 2020; Rawlings and Legare, 2020). The questionnaire is mainly composed of three parts, namely, the questionnaire for the working environment, the questionnaire for innovation behavior, and the questionnaire for business performance. The questionnaire includes the basic information of the tested personnel, the type of innovation selected by the tested personnel, the management means of enterprise managers, the percentage of enterprise developers, the establishment time of the enterprise, and the industry in which the enterprise is located. Questionnaire results are analyzed using Statistical Product and Service Solutions 25.0, as shown in Table 1.

**Table 1:** Main composition of questionnaires for employees.

**Classification of influencing factors**	**Composition of main indexes**	**Main content**
Innovative behavior	Exploratory innovation	Bring new products to enterprises.
		Expand the scope of business products.
		Make enterprises enter new markets.
	Applicability innovation	Improve the product quality for enterprises.
		Improve the flexibility and productivity of existing products.
		Save production costs of existing products.
	Innovative ways	Strive for resource support for realizing new conception.
		Formulate appropriate innovative plans.
	Innovation willingness	Make employees who seek innovation actively.
		Convince others to work efficiently.
		Discover new technologies.
Working environment	Management means	Improve employees' enthusiasm.
		Make employees more expressive.
		Provide good conditions for employees' innovation.
	Policy impact	Gain innovation grant from the government.
		Obtain policy support from the government.
		Get hardware support from the government.
	Economic system	Get national macro-economic system support.
		Get the support from the Enterprise ownership and organization form.
	Market development	Obtain a sound and effective market for enterprises.
	Legal environment	Have a legal enterprise environment.
	Office environment	Create a comfortable office environment for employees.
Others	Performance of new ventures	Achieve enterprise profits.
		Take up market shares of enterprises in the industry.
		Increase the growth rate of enterprise sales.

Table 1 shows that the questionnaire includes different characteristics of respondents. Since the policies of different cities are different, the comparison between the indicators items and competitors is added in the same industry to reduce the differences brought by different cities and make the results more universal. The basic information helps to determine the characteristics of the surveyed enterprises and employees and is conducive to the development of the following research.

A city is selected as the research object, and the questionnaires are distributed offline and online. The offline questionnaires are mainly distributed to major enterprises, and the online and offline data are collected, with 500 copies and 420 valid questionnaires. An online questionnaire investigation is conducted on 1,500 employees, and the valid questionnaires are 1,273 copies. A total of 2000 questionnaires are distributed, and 1,693 valid questionnaires are collected, with a total recovery rate of 84.7%. In the questionnaires, the invalid questionnaires are those whose answers to questions in the questionnaire are <15%.

### Test Method of Questionnaire Data

Since the data of the structural equation model must be tested, it is necessary to test the reliability and validity of the questionnaire. In this study, statistical Product and Service Solutions software is used for data statistical tests. First, descriptive statistical analysis is carried out to verify the standard deviation, mean, and variance of the experimental data. The kurtosis of each datum is not more than 10, and the data offset is not more than 3, indicating that the data meets the standard (Kottwitz et al., 2019). The 789 data about the work environment and 904 data about innovation behavior are statistically analyzed. The reliability and validity test of this study are tested by KMO (Kaiser-Meyer-Olkin). According to Butterlit spherical test, if the KMO data are between 0.7 and 0.8, the data are available; If it is >0.8, the data are good. Based on the internal consistency coefficient, the reliability of the data is calculated by the software. When the coefficient is >0.7, the data reliability is good. After the conditions are satisfied, factor decomposition is carried out by SPSS software, and the minimum factor load of the abstract variable spindle is >0.5, which shows that the data validity is good and meets the requirements of the structural equation model.

## Analysis of Influencing Factors of New Ventures

The specific results of the basic information are shown in Table 2. Based on the questionnaire survey results, the basic information data of enterprise employees are analyzed.

**Table 2:** Statistical results of specific information of respondents.

**Items**	**Options**	**Percentage (%)**
Age	<24 years	7.59
	25–36 years	58.91
	37–46 years	24.67
	>47 years	8.83
Gender	Female	35.7
	Male	64.3
Innovation types	Applicability innovation	54.68
	Exploratory innovation	45.32
Education	Bachelor's degree or below	45.75
	Master's degree	43.49
	Doctor's degree or above	10.76
The percentage of enterprise developers	<8% 8–16%	39.79 45.17
	More than 16%	15.04
Number of employees	<150	49.10
	150–250	30.98
	More than 250	19.92
The establishment time	<3 years	30.79
	3–7 years	49.89
	More than 7 years	19.32

Table 2 shows that the basic information of employees in this survey shows that the proportion of people under the age of 24 is 7.59%, and the proportion is the least in all age groups. The age group from 25 to 36 is the most, indicating that employees in enterprises are mainly young people. Female employees account for 35.7%, far less than the number of male employees, which is consistent with the proportion of males and females in China's employment market (Kenett and Faust, 2019; Meagher, 2020; Holm-Hadulla et al., 2021). The number of people who choose applicability innovation is much larger than the number of people who choose exploratory innovation. The analysis shows that the demand for product performance is the driving force to promote employees' innovative behavior. The proportion of the people having a bachelor's degree or below is the largest, which is 45.75%, and the proportion of the people with a doctor's degree is the least. This shows that employees have a bachelor's degree or below. The survey of basic information shows that the proportion of enterprise developers is between 8 and 16%, and the proportion of enterprise developers is relatively small. The number of employees in enterprises is <150, the number of the enterprise having 250 employees is the least, with a percentage of 19.92%. The establishment time of the surveyed enterprises is 3 to 7 years. The proportion of enterprises with establishment time <3 years is 30.79%, and the proportion of enterprises with an established time of more than 7 years is 19.32%.

## Descriptive Statistical Analysis and Reliability and Validity Test

### Descriptive Statistical Analysis

The average characteristics of each questionnaire datum are shown in Table 3.

**Table 3:** Average characteristics of questionnaire data.

**Types of questionnaire**	**Data**	**Mean**	**Bias**	**Standard deviation**	**Kurtosis**	**Maximum data offset**
Working environment	789	3.9056	−0.9019	1.0754	0.535	2.830
Innovation behavior	904	3.9209	−0.8712	0.9891	0.479	2.019

According to the means of the statistical data in Table 3, the maximum data deviations of each datum are 2.830 and 2.019, which are <3, and the kurtosis of each datum is <3. The means of all data are about 3.9. From the numerical relationship between standard deviation and means, it is found that the data are concentrated and there are no extreme data. Thus, the test results of the data of the questionnaire are good. There are no abnormal questionnaire data, indicating that the questionnaire data are credible.

### Reliability and Validity Test

The validity test data of this study are shown in Table 4.

**Table 4:** Results of validity test.

**Types of questionnaire**	**KMO**	**Bartlett test of sphericity**			**Minimum number of loads of principal axis factor of an abstract variable**
		**df**	**Sig**.	**Approximate chi-square**	
Working environment	0.891	476	0.000	6,123.738	0.621
Innovation behavior	0.857	570	0.000	1,1163.523	0.583

The data in Table 4 show that the KMO values of questionnaire data are 0.891 and 0.857, which are >0.8, indicating that the data are good and can be tested by factor analysis. The minimum load number of spindle factor of the abstract variable is 0.583 > 0.5, indicating good data validity.

The above shows that this study contains 11 abstract variables and 25 observable variables. The abstract variables are managerial influence (GL), policy impact (ZC), economic system (JJ), market development (SC), legal environment (FZ), office environment (BG), exploratory innovation (TS), applicability innovation (SY), innovation implementation (SX), innovation willingness (YY) and new venture performance (JX). SPSS 25.0 software is used for reliability tests, and the results are shown in Table 5.

**Table 5:** Test results of reliability.

**Abstract variables**	**Internal consistent coefficients**	**Observational variables**	**Internal consistent coefficients**
GL	0.902	GL1	0.832
		GL2	
ZC		ZC1	0.872
		ZC2	
		ZC3	
JJ		JJ1	0.838
		JJ2	
SC		SC1	0.857
FZ		FZ1	0.799
BG		BG1	0.872
TS		TS1	0.851
		TS2	
		TS3	
SY		SY1	0.798
		SY2	
		SY3	
SX		SX1	0.861
		SX2	
YY		YY1	0.819
		YY2	
		YY3	
JX		JX1	0.869
		JX2	
		JX3	

*The alphabetic initials of influencing indicators are selected to replace Chinese characters*.

Table 5 shows that the internal consistency coefficient is between 0.798 and 0.902, and the minimum value is 0.798 >0.7. Therefore, the data reliability of this study is good, which meets the requirements of the structural equation model. This shows that the data of the questionnaire are credible and the data can be analyzed as a whole.

### Correction of the Structural Equation Model

**Figure 3:**
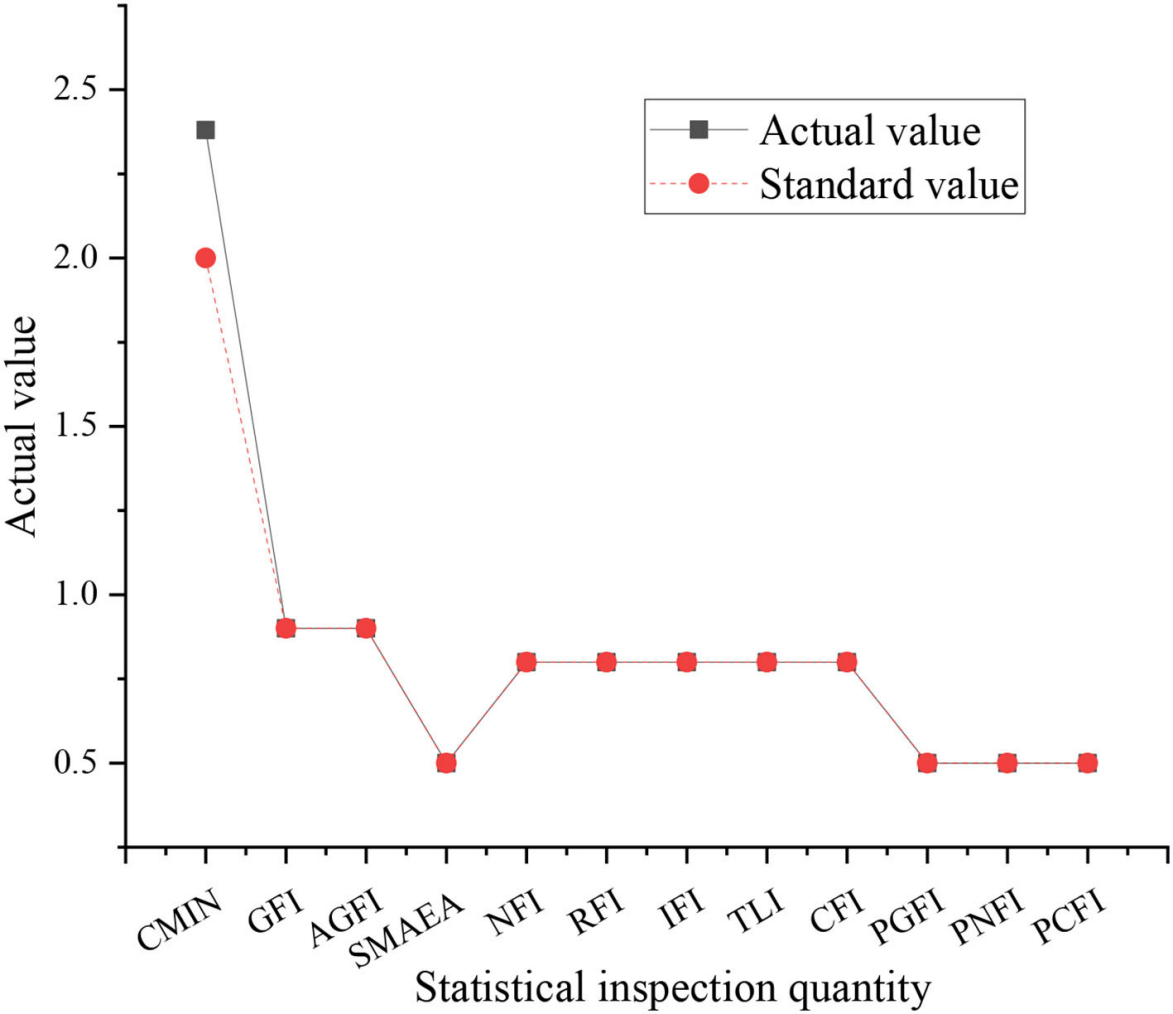
Software simulation results.

The model modification in this study is carried out by AMOS software, and the significance of the model is good when the significance probability is <0.05. The results show that the significance (Sig.) of the impact model of the working environment and innovation behavior on business performance is 0.000, and the model meets the requirements, but the number of samples is too large. In this case, the significance probability cannot accurately classify the good grades of the model. Therefore, the chi-square- ratio of freedom degree and other indicators are used to explore the advantages and disadvantages of the model. The fitting results of the software simulation are shown in Figure 3.

**Figure 4:**
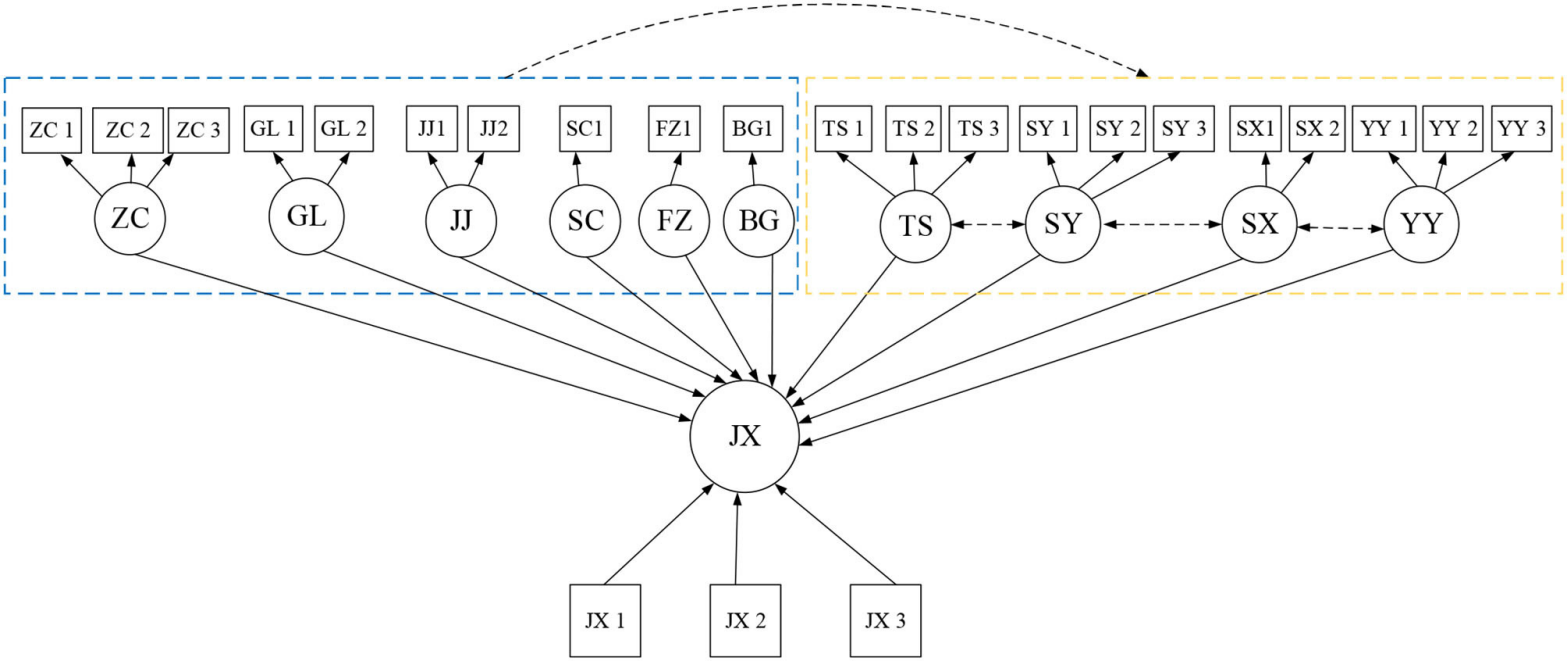
Optimized model.

Figure 3 shows that among the 12 data comparisons, 11 of them are consistent with the actual data, and the results of software simulation are good. However, there is a data of 2.0 that does not match the actual value of 2.5. The data belong to the absolute adaptation index, and there is no mismatch phenomenon. Therefore, the model needs to be corrected, otherwise, it will lead to serious errors in model analysis. Therefore, the above correction method is used. Starting from the simplified model and the actual aspects, the model of the impact of the working environment and innovation behavior on corporate performance is optimized and modified. The correction results are shown in Figure 4.

**Figure 5:**
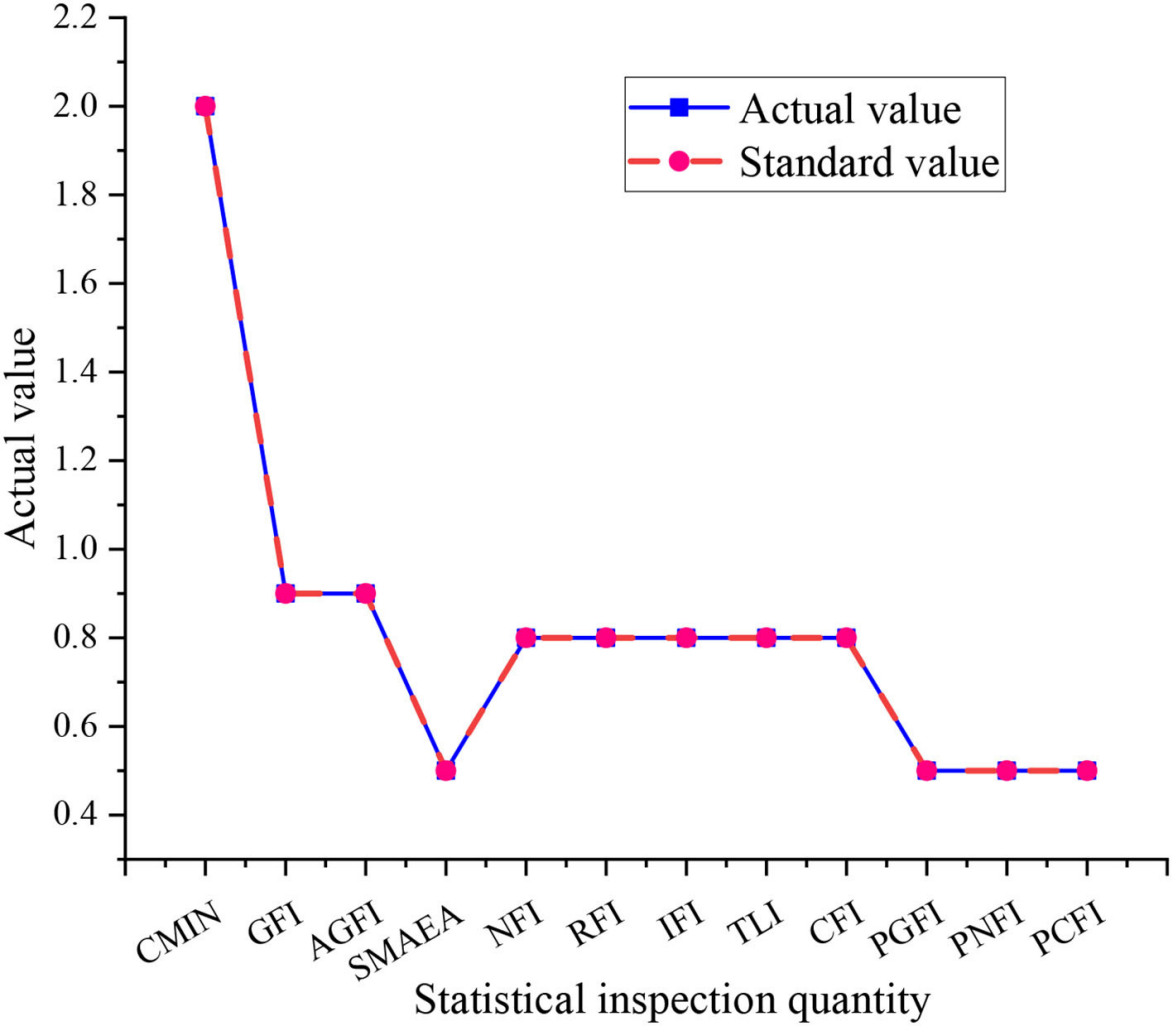
Fitting results of the optimized model.

The optimized model in Figure 4 shows that the optimized model is more complete, and each abstract index adds specific influencing factors. After optimization, the influence effect between each index is added, so that the whole model structure is more viscous. This study aims to explore the relationship between the influencing factors. The establishment of Figure 4 facilitates the discussion of the correlation between the influencing factors involved in this study. The research purpose of this paper will be discussed in detail based on Figure 4. The optimized model is tested, and the results are shown in Figure 5.

**Figure 6:**
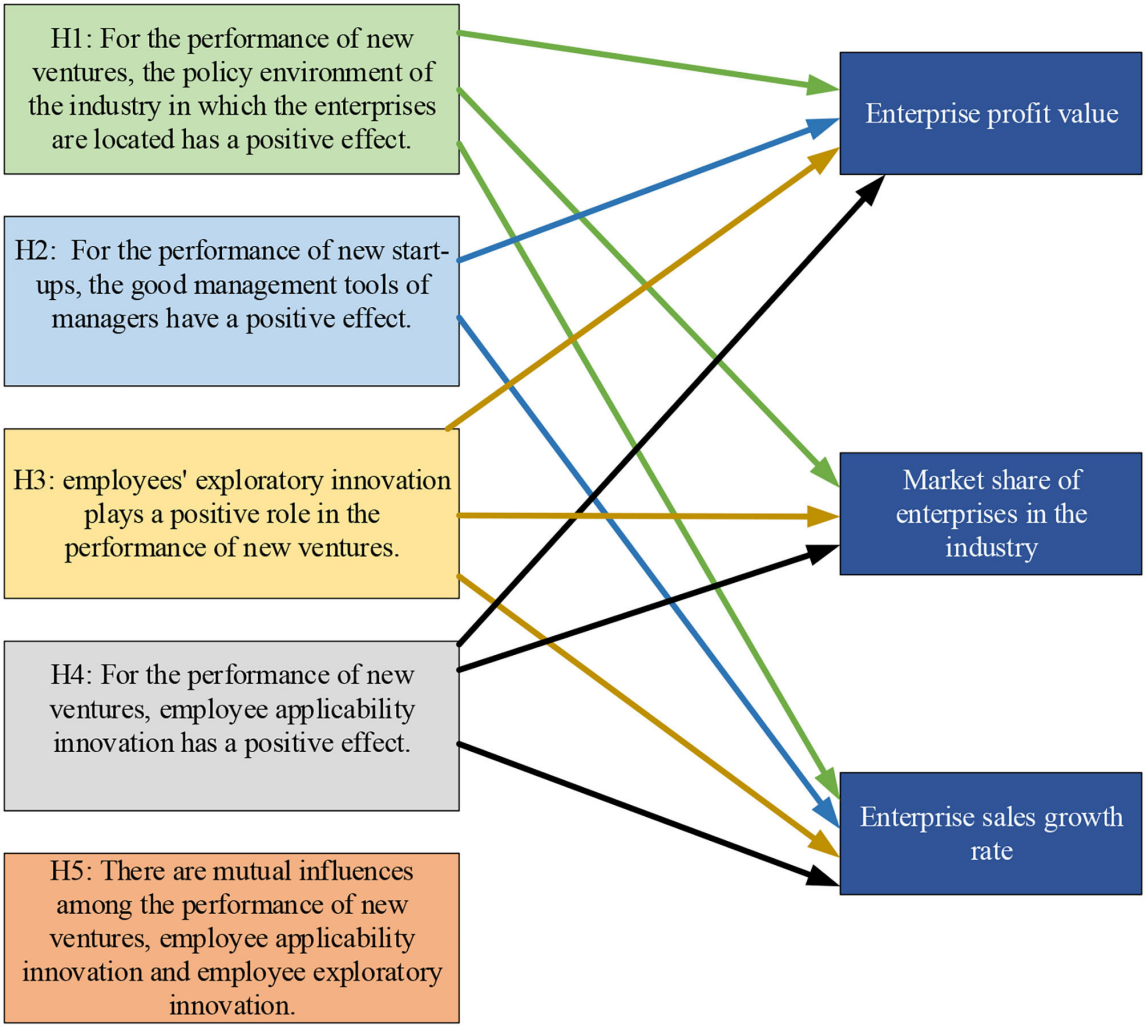
Block diagram of hypothetical results of various influencing factors.

Figure 5 shows that the optimized model has good data, 12 data are consistent with the actual data, no abnormal data, and data need to be corrected. Since the path coefficient in the previous model is set to be 1, the modified path coefficient is calculated by the software, and the results show that the optimized path coefficient is 0.012. Based on the results of the above optimization model data, the hypotheses above are studied. The establishment of the hypotheses is shown in Figure 6.

The hypothesis results in Figure 6 show that the policy environment of the industry where the new venture is located affects the corporate performance by influencing the corporate profitability, the market share of the enterprise in the industry, and the growth rate of corporate sales. Managers' management ability is directly related to corporate profitability and the growth rate of corporate sales. Employees' exploratory innovation behavior and applicability innovation behavior can promote enterprise profit value, enterprise market share in the industry, and the growth rate of corporate sales. There is no interaction between employee applicability innovation and exploratory innovation, so hypothesis 5 is not valid. It can be concluded that among the five hypotheses proposed at the beginning of this study, the first to fourth hypotheses are valid, and the fifth hypothesis is not valid. That is to say, the corporate performance of new ventures is affected by the policy environment, the management mode of managers' behavior, but there is no interaction between employees' exploratory innovation behavior and applicability innovation behavior. The reason may be that employees' information communication is not timely, and exploratory innovation and applicability innovation cannot be used by each other.

**Figure 7:**
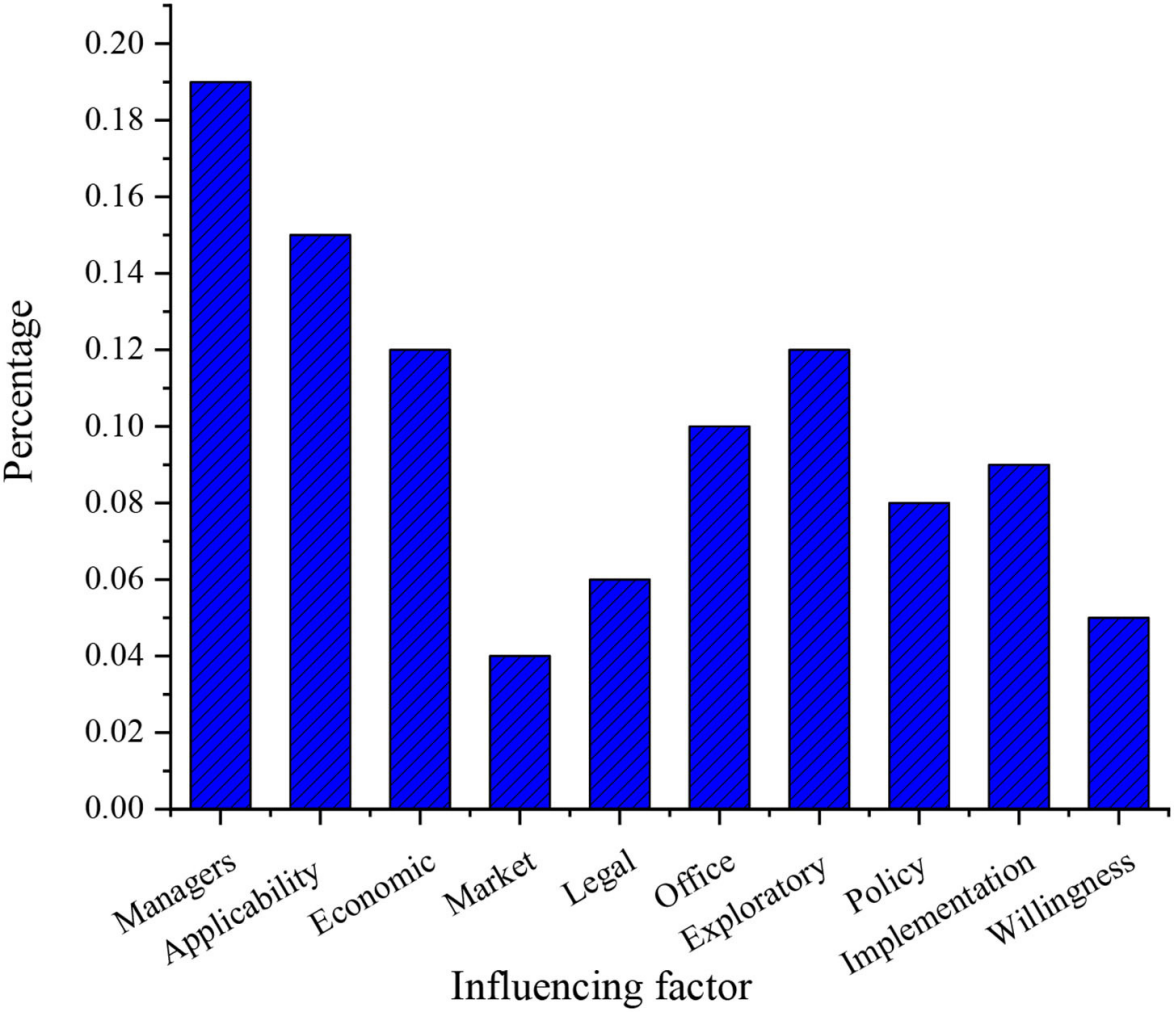
Results of the proportion of various factors.

The influence of each specific factor on the enterprise indicator is shown in Figure 7. The influence of the index on the enterprise profit value, the market share of the enterprise in the industry, and the growth rate of enterprise sales are unified as the influence on enterprise performance to simplify the problem.

Figure 7 shows that good management practices of managers have the greatest impact on corporate performance, accounting for 19 %, which is consistent with the research results of Zhou and Wu (2018) that employees often show positive work emotions when facing humble managers (Zhou and Wu, 2018). Employees' applicable innovation behavior accounts for a small proportion of 15%. Employees' exploratory innovation behavior accounted for 12%. The policy environment in the industry has the least impact on corporate performance, accounting for 8%. The reason may be that the manager's management directly affects the enthusiasm of employees and corporate performance. The comprehensive proportion of employees' two innovative behaviors reaches 27%. This shows that employees' innovative behaviors have a great impact on enterprise performance, and the impact of employee exploratory innovation on enterprise performance is slightly lower than that of employee applicability innovation. The reason may be that applicability innovation is selected by more employees who pay more attention to solving the problems encountered in work. Exploratory innovation is difficult to succeed in, and it has little impact on corporate performance. In addition, the impact of the economic system on corporate performance accounts for 12%. The economic system affects corporate performance by affecting employees' work behavior, and other influencing factors have <10% impact on corporate performance. The minimum proportion of policy impact on enterprise performance is because most of the enterprises in this survey are new ventures, and their market share in their respective fields is small, and they have not yet formed a mature organizational structure.

## Discussion

The impact of the work environment and innovation behavior of new ventures on corporate performance is analyzed and discussed by setting 11 abstract variables and the results of the questionnaire survey based on personality psychology. Based on five abstract variables and the results of the questionnaire survey, the impact of the work environment and innovation behavior of new ventures on business performance from the perspective of personality psychology are analyzed and discussed. The results of the questionnaire first display the basic information of the respondents and enterprises, which laid a good foundation for the construction of the model. Most of the establishment time is <7 years, and enterprise personnel mainly have bachelor's degrees. Compared with other factors, the management of managers also has an impact on business performance. Zhou and Wu (2018) argued that employees often had positive attitudes when their managers are humble, which is proved in the study. Based on the two perspectives of the working environment and innovation behavior, five hypotheses are put forward. Based on 11 abstract variables, 25 observable variables are proposed and the corresponding structural equation model is built.

Based on the structural equation model, the impact of new ventures' working environment and innovation behavior on corporate performance. The indexes of the model meet the test standards. The model fits well with the measured data, indicating that the model can be used for the study. In the model, 10 influencing factors of new ventures have different effects on corporate performance, and they are the management mode, policies, the economic system, market development, the legal environment, the office environment, exploratory innovation, applicability innovation, the innovation realization mode, innovation intention, and new venture performance. Among them, the management mode of managers has the greatest impact on corporate performance, accounting for 15%, followed by the impact of applicability innovation and the impact of the economic system. Policies and exploratory innovation have little impact on corporate performance. The analysis results may be because the enterprises involved in this survey are all new ventures and have not reached the standard for the care of policies. Exploratory innovation is too difficult, so the probability of success is small.

The hypothesis results of this study show that exploratory innovation and applicability innovation have no influence on each other, and the reason may be the inconsistency of information transmission, which makes the innovation results not be used each other. In summary, the working environment and innovation behavior has a huge impact on corporate performance.

## Conclusion

The influencing factors of the performance of new ventures and the structural equation model are introduced to analyze the influence of enterprise environments and innovation behavior on corporate performance to improve the performance of new ventures, and finally, a questionnaire is designed to analyze the influencing factors of new ventures in detail. The modified model in this study has good performance. The results of modeling analysis show that the 10 influencing factors of new ventures in the model have different effects on corporate performance, and they are the management mode, policies, the economic system, market development, the legal environment, the office environment, exploratory innovation, applicability innovation, the innovation realization mode, innovation willingness and the performance of new ventures. Among them, the management mode of managers has the greatest impact on enterprise performance, followed by applicability innovation and the economic system. Policies and exploratory innovation have little impact on enterprise performance. The hypothesis results of this study show that among the five hypotheses proposed in this study, the first four hypotheses are valid, and the fifth hypothesis is not valid. That is to say, the corporate performance of new ventures is affected by the policy environment, managers' management methods, and employees' innovation behaviors, but exploratory innovation and applicability innovation do not influence each other. It is speculated that the reason may be the inconsistency in information transmission so that the innovation results are not used mutually. This study explores the influencing factors of the performance of new ventures, which has certain positive significance for new ventures to improve their corporate performance based on working environment and innovation behavior. However, the shortcoming of the study is that a few representative indexes for the working environment and innovation behavior are only selected, resulting in one-sided research results. Because of this, in future research, more indexes should be set to make the research results more accurate and universal. The research results provide a reference for the improvement of the corporate performance of new ventures and a direction for enterprises to improve corporate performance through the construction of working environment and innovation behavior.

## Data Availability Statement

The raw data supporting the conclusions of this article will be made available by the authors, without undue reservation.

## Ethics Statement

The studies involving human participants were reviewed and approved by Zhejiang University of Science and Technology Ethics Committee. The patients/participants provided their written informed consent to participate in this study. Written informed consent was obtained from the individual(s) for the publication of any potentially identifiable images or data included in this article.

## Author Contributions

All authors listed have made a substantial, direct, and intellectual contribution to the work and approved it for publication.

## Conflict of Interest

The authors declare that the research was conducted in the absence of any commercial or financial relationships that could be construed as a potential conflict of interest.

## Publisher's Note

All claims expressed in this article are solely those of the authors and do not necessarily represent those of their affiliated organizations, or those of the publisher, the editors and the reviewers. Any product that may be evaluated in this article, or claim that may be made by its manufacturer, is not guaranteed or endorsed by the publisher.
